# A GAN-based anomaly detector using multi-feature fusion and selection

**DOI:** 10.1038/s41598-024-52378-9

**Published:** 2024-03-04

**Authors:** Huafeng Dai, Jyunrong Wang, Quan Zhong, Taogen Chen, Hao Liu, Xuegang Zhang, Rongsheng Lu

**Affiliations:** 1 Tsinghua University, Beijing, China; 2 LCFC (Hefei) Electronics Technology Co., Ltd., Hefei, Anhui China; 3 Hefei LCFC Information Technology Co., Ltd., Hefei, Anhui China; 4 Hefei University of Technology, Hefei, Anhui China

**Keywords:** Computational science, Computer science

## Abstract

In numerous applications, abnormal samples are hard to collect, limiting the use of well-established supervised learning methods. GAN-based models which trained in an unsupervised and single feature set manner have been proposed by simultaneously considering the reconstruction error and the latent space deviation between normal samples and abnormal samples. However, the ability to capture the input distribution of each feature set is limited. Hence, we propose an unsupervised and multi-feature model, Wave-GANomaly, trained only on normal samples to learn the distribution of these normal samples. The model predicts whether a given sample is normal or not by its deviation from the distribution of normal samples. Wave-GANomaly fuses and selects from the wave-based features extracted by the WaveBlock module and the convolution-based features. The WaveBlock has proven to efficiently improve the performance on image classification, object detection, and segmentation tasks. As a result, Wave-GANomaly achieves the best average area under the curve (AUC) on the Canadian Institute for Advanced Research (CIFAR)-10 dataset (94.3%) and on the Modified National Institute of Standards and Technology (MNIST) dataset (91.0%) when compared to existing state-of-the-art anomaly detectors such as GANomaly, Skip-GANomaly, and the skip-attention generative adversarial network (SAGAN). We further verify our method by the self-curated real-world dataset, the result show that our method is better than GANomaly which only use single feature set for training the model.

## Introduction

In recent years, the anomaly detection task has become increasingly important within many domains, such as visual image understanding, medical applications^1^, and industrial anomaly detection^2^. Anomaly detection refers to the problem of finding patterns in data that do not conform to expected behavior^3,4^. The definition of normal samples and abnormal samples depend on the specific task. For example, in the electronics manufacturing, a product with no defect is so called a normal sample, and a product with defects is called an abnormal sample. Similarly, in the finance field, fraud detection task, a customer with no fraud record is a normal sample, and a customer with fraud records is an abnormal sample, and so on. In more general definition, a sample in the distribution is a normal sample, and a sample out of the distribution is an abnormal sample also known as an outlier in some fields.

Supervised methods can achieve good results when large datasets are available^5–9^. However, in most cases, such data resources are hard to collect, and the lack of abundant abnormal samples caused the available dataset to be highly imbalanced. Furthermore, the available abnormal samples may offer only partial coverage of all possibilities. Hence, under the condition that these datasets lack capacity and diversity, it is difficult to train a supervised model to have good performance. For handling such scenarios, unsupervised anomaly detection methods have been proposed. These methods are trained only by normal samples to learn the normality distribution based on abundant normal samples, and then these methods use the deviation derived from the distribution to distinguish abnormal samples from normal samples.

There are several unsupervised approaches to handle the anomaly detection task^10,11^, including but not limit to the embedding-based methods^12^, generative adversarial network (GAN)-based methods^13–16^, reconstruction-based methods^17^. The anomaly detection methods can capture the information from the majority class. Despite these methods can also use the abnormal data to capture its information and can distinguish normal data from the deviation. However, it is hard to collect sufficient abnormal data in the real-world situation. Recently, segmentation-based methods achieve good performance on the MVTec AD dataset such as DeSTSeg^18^ and MemSeg^19^. However, the labeled masks which used for training a segmentation network is hard to collect. In our previous study^20^, we have collected the LCFC-Laptop dataset for surface defect detection by using object detection manner to label this dataset, i.e., we only labeled the bounding boxes to save the labelling effort. It still costs more than one year to collect approximately 2500 pieces laptop shells. Despite labelling the bounding box is far easier than labelling a segmentation mask, the noisy label is still inevitable. Some methods ^21,22^ use extra datasets for training to improve the prediction performance. These authors develop a customized feature adaptor for the target dataset (MVTec AD). However, the feature adaptor seems not suitable for all dataset. SimpleNet achieves 99.6% AUC on the MVTec AD dataset and achieves only 86.5% AUC on the CIFAR-10 dataset. When the same adaptor applies to another dataset, the AUC performance is decreased. There is an extended question to select one or more good dataset(s) which have better performance to adapt to the interested dataset. Another question is how to design a general purpose “adaptor” module which can easily applied to numerous datasets without significant modification with good performance. Some methods such as OCR-GAN ensembles multiple models to achieve a good performance on the MVTec AD dataset. Wave-GANomaly compared to the ensemble methods only used one model which is easier to deploy. These methods have a common hypothesis that abnormal samples differ from normal samples in both image space and latent space encoding. Hence, it is critical to obtain the distribution of normal samples. The embedding-based methods designed by considering the error of latent space with lightweight design. The reconstruction-based methods by considering the reconstruction error between the normal samples and abnormal samples. The GAN-based methods by simultaneously considering the reconstruction error and the latent space error. Fortunately, recent developments in GAN-based methods have shown the ability to capture input data distributions^13^. These GAN-based methods also have a good ability to map the image space to latent space and vice versa with minimal information loss^15,16^.

This prior work in the field^14–16^ empirically illustrates both the importance and promise of anomalies within dual image and latent space. Akçay et al.^15^ trained an encoder-decoder-encoder network, GANomaly, with the adversarial scheme to capture the normal distribution within the image and latent space. Akçay et al.^23^ proposed a method, Skip-GANomaly, for anomaly detection via adversarial training over a skip-connected encoder-decoder (convolutional neural) network architecture. Adversarial training has shown the promise of GANs in this domain^15^. Liu et al.^24^ proposed a method called skip-attention generative adversarial network (SAGAN), which is based on the GANomaly and Skip-GANomaly methods, using the convolutional block attention module (CBAM) ^25^ to improve their performance on the anomaly detection task. On the high-level design of anomaly detectors, this elegant GANomaly research compared various model designs including the variational autoencoder (VAE)^17^, AnoGAN^26^, and efficient GAN-based anomaly detection (EGBAD)^16^. VAE uses the encoder for modeling the distribution of the latent variables rather than the latent variable itself. The authors of VAE assume that abnormal samples will have greater variance and a lower probability of successful reconstruction than normal samples. The generator *G* of AnoGAN learns a distribution over data *x* via a mapping *G*(*z*) of samples *z*, 1D vectors of uniformly distributed input noise sampled from latent space* Z*, to 2D images in the image space manifold X, which is populated by normal samples. The AnoGAN model design is based on uniformly distributed input noise. EGBAD is based on the bidirectional generative adversarial network (BiGAN)^27^, which simultaneously learns an encoder that maps input samples *x* to a latent representation, along with a generator *G* and discriminator* D* during training. Among the four high-level model design architectures are GANomaly based, VAE, AnoGAN and EGBAD style designs. The GANomaly based architecture outperforms the other compared designs. Hence, the present work is also founded on the GANomaly based architecture and explores multimodal features from the wave-based feature set and convolutional-based features for the anomaly detection task and evaluates them with the Canadian Institute for Advanced Research (CIFAR)-10^27^ and Modified National Institute of Standards and Technology (MNIST) datasets^28^.

These GAN-based models use only single feature set to capture the information of the input data and explore various model design architectures on many applications. However, the ability to capture the information of each feature set is limited. The direct fusion of different feature sets causes high dimension features which led to high computation cost. Furthermore, not all feature equally contributed to the interested task. Hence, it is important that good combination use of fusion and selection of feature sets.

Wave-based features are extracted by WaveBlock^29^, which decomposes a given input into various phases and amplitudes of basic waves. A WaveBlock module proposed by Huawei, Peking University, and other units from the Computer Vision and Pattern Recognition Conference (CVPR) 2022 are an improvement of the multilayer perceptron (MLP) module. It is a quantum-inspired design and achieves good performance on 2D classification, 2D object detection and 2D object segmentation tasks^29^. Wave-MLP decomposes the input into different de Broglie waves with different phases and amplitudes. In other words, a given input can be regarded as a combination of basic waves with different phases and amplitudes.

In this work, we explored better feature fusion and selection from wave-based and convolution-based feature sets. For the sake of fair comparison, we use the same hyperparameter settings as those used in Skip-GANomaly. We propose a model, Wave-GANomaly, by fusing and selecting from the wave-based features and the convolution-based features. The model design is similar to the designs of GANomaly, Skip-GANomaly, and SAGAN. The U-Net style design^30^ is known to capture the distribution of input in a multiscale manner. Despite the original U-Net style network is designed for the segmentation task, the differences among Wave-GANomaly and the original U-Net are listed below. First, the output of Wave-GANomaly is the reconstruction image of a given input image instead of the classification map which is the original output to each pixel. The second difference between Wave-GANomaly and U-Net style networks is the loss function. Wave-GANomaly considers embedding loss and reconstruction loss at a time. The U-Net style networks for segmentation usually use the classification error to the labeled masks. Furthermore, Wave-GANomaly trained in an unsupervised way instead of supervised way which adopted by the U-Net style segmentation network.

The contributions of this work can divide into several aspects. First, compared methods which use various extra modules designed for a specific dataset^22^, this work is an end-to-end approach. Second, compared methods which use large amount of training parameters, this work uses a lightweight design. In fact, numerous scenes have strict limit to the prediction time. Hence, an accurate but slow method may not fulfill the requirements of these scenes. Third, this work trained in an unsupervised way which means the effort of labeling data can significantly reduce to a very low level. Recently, multi-modal studies which extract multi-feature from various types of data have achieve great success in many applications. For example, CLIP^31^ proposed by OpenAI uses features from image and text achieves completive performance to the supervised methods. However, simply fuse various sets of features may have negative impacts on the prediction. Hence, this work first fuses and selects features from multiple features, improving the performance of these studies which only use single set of features.

We evaluated Wave-GANomaly using the benchmark datasets CIFAR-10^32^ and MNIST^33^ by performing leave-one-class-out experiments. The evaluations showed that Wave-GANomaly outperforms existing state-of-the-art methods in terms of the average area under the curve (AUC).

## Materials and methods

This section presents an overview of the datasets, the design of the experiments and the design of Wave-GANomaly for introducing the experimental design and setting and also provides a brief review of the baseline method (Skip-GANomaly). Each module of Wave-GANomaly, the used objective functions, and the calculation of the abnormal score for quantifying the given input tend to be a normal sample or an abnormal sample from the deviation of the well-trained model.

### Datasets and design of experiments

#### The CIFAR-10 dataset

The CIFAR-10 dataset is a collection of images that are commonly used to train machine learning models on computer vision tasks. The CIFAR-10 dataset contains 60,000 color images in 10 different classes. The 10 classes are airplanes, automobiles, birds, cats, deer, dogs, frogs, horses, ships, and trucks. There are 6,000 images of each class. Experiments for the CIFAR-10 dataset use the leave-one-class-out approach to conduct related experiments. In a given experiment, one class is regarded as containing abnormal samples, and the other classes are regarded as containing normal samples. When testing our models, we adopt a ratio of 3:2 (normal to abnormal) to test our models. The experimental design of our model is the same as that of the GANomaly and Skip-GANomaly models.

#### The MNIST dataset

The MNIST dataset is a large dataset of handwritten digits that is commonly used for training various image processing systems. The MNIST dataset contains 60,000 training images and 10,000 test images^34^. There are 10 classes in the MNIST dataset: 0, 1, 2, 3, 4, 5, 6, 7, 8, and 9. Similarly, experiments on the MNIST dataset (GANomaly^15^) are conducted by regarding one class as containing abnormal samples, while the rest are considered classes containing normal samples. In total, we have ten sets of data, each of which considers individual digits as abnormal samples.

#### The LCFC-laptop-defect dataset

LCFC (Hefei) Electronics Technology Co., Ltd. (LCFC) is a wholly owned subsidiary of Lenovo. Worldwide, one out of eight laptops sold are manufactured by LCFC; the cumulative number of laptops sold has reached 0.2 billion, and over 126 countries have bought laptops from Lenovo. Hence, through collaboration with this company, we were able to gain the opportunity to establish an extensive laptop dataset to verify the performance of Wave-GANomaly.

To construct this dataset, we collected samples acquired by four 5000 × 5000 high-resolution industrial cameras with 6 sets of lights, including white lights, blue lights, and red lights. We believe that a given defect is associated with a combination of specific wavelengths and that illumination with a broad spectrum can allow a variety of defects to be well captured. Hence, we used various wavelengths of light to collect this dataset. The 6 sets of lights were tuned by senior optical engineers. Next, we used the same standards adopted by the senior engineers and quality inspectors of LCFC to label this dataset. For fitting the Wave-GANomaly input requirements and saving computation time, we extract a square region which with 128 × 128 resolution around the defect from the original 5000 × 5000 image. We also extract normal region with the same 128 × 128 resolution from the original 5000 × 5000 image. Finally, we obtained a dataset consisting of 2,091 normal samples and 1,157 abnormal samples. Although the dataset was labeled by senior engineers and quality inspectors, some mislabeled samples are inevitable. The mislabeled samples can be divided into three categories. First, a sample of one class may be labeled as belonging to another class. Second, a nondefect sample may be labeled as a defect sample. Third, a defect sample may be labeled as a nondefect sample. Since the samples in this dataset will be on the market within 6 months to 12 months, this dataset analyzed during the current study available from the corresponding author on reasonable request.

### The design of wave-GANomaly

#### Skip-GANomaly as the baseline model

The elegant proposal Skip-GANomaly^23^ adversely trained the encoder-decoder architecture for unsupervised learning from normal samples only, while Skip-GANomlay tested both normal and abnormal samples. In practical settings, the number of normal samples is far larger than the number of test samples. A high-level overview of this model consists of a generator (*G*) and a discriminator (*D*). G adopts the bow-tie design by an encoder (*G*_*E*_) and a decoder (*G*_*D*_). The encoder aims to capture the distribution of input images by finding a good mapping function from the high-dimensional input images to the low-dimensional latent space. This work is an improved version of another elegant proposal, GANomaly^15^. It improved the GANomaly model by adding skip connections to integrate the information from the encoder instead of using only the decoder information (GANomaly). GANomaly and Skip-GANomaly provide source code and flexible design to allow the research community to customize their model according to their own dataset. Another elegant proposal, SAGAN^24^, improved Skip-GANomaly by adding a CBAM^25^, which is an attention module obtained by mixing the spatial and channel attention modules. As a result, SAGAN outperforms Skip-GANomaly. However, SAGAN source code is not available in the public domain. Hence, in this work we reimplemented SAGAN to fairly compare these state-of-the-art methods on the benchmark dataset by means of an ablation study.

#### The design of wave-GANomaly

**Figure 1:**
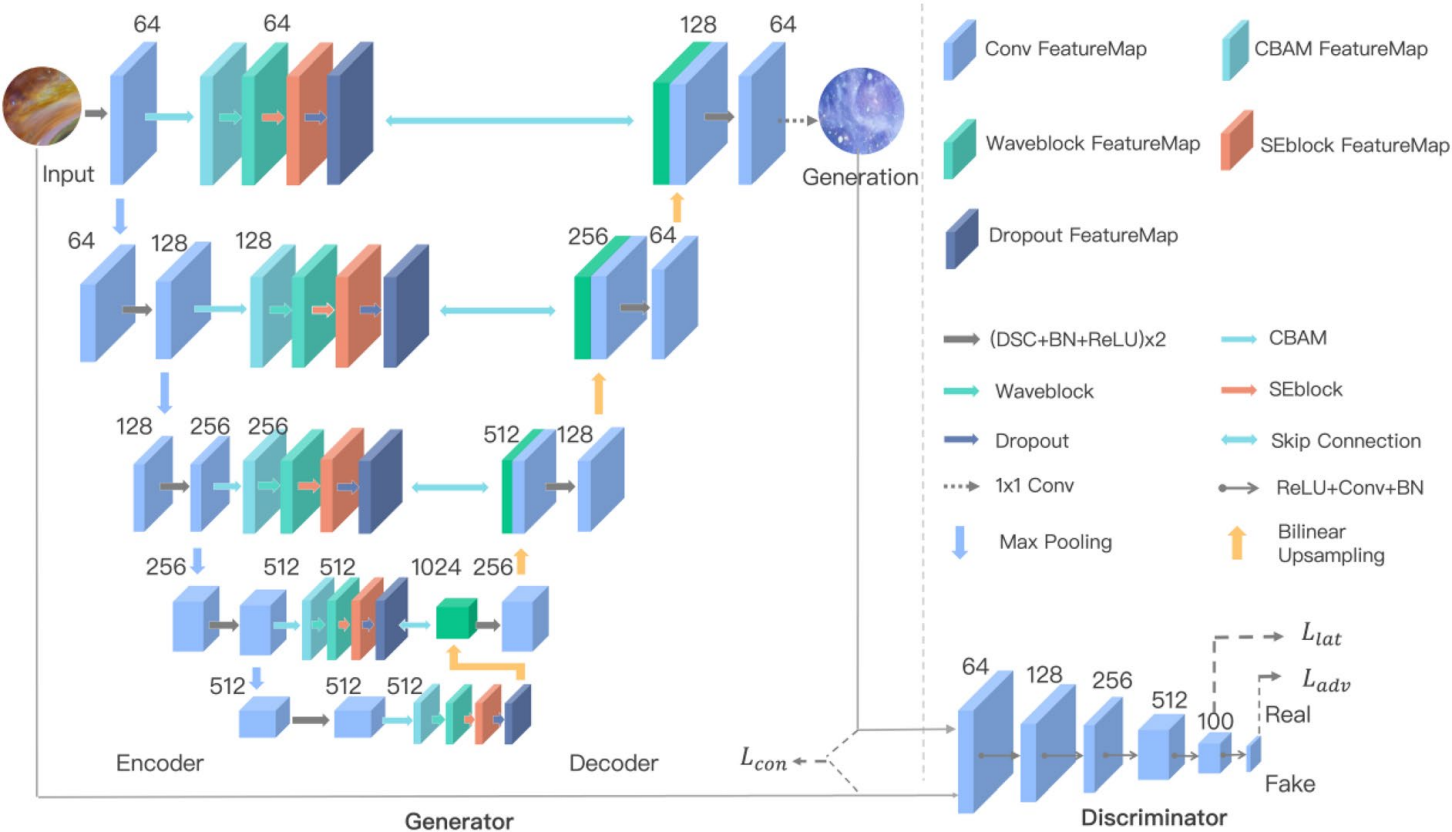
The design of Wave-GANomlay. Wave-GANomaly is designed by integrating Wave-Block, SE-Block, CBAM, and DropOut.

**Figure 2:**
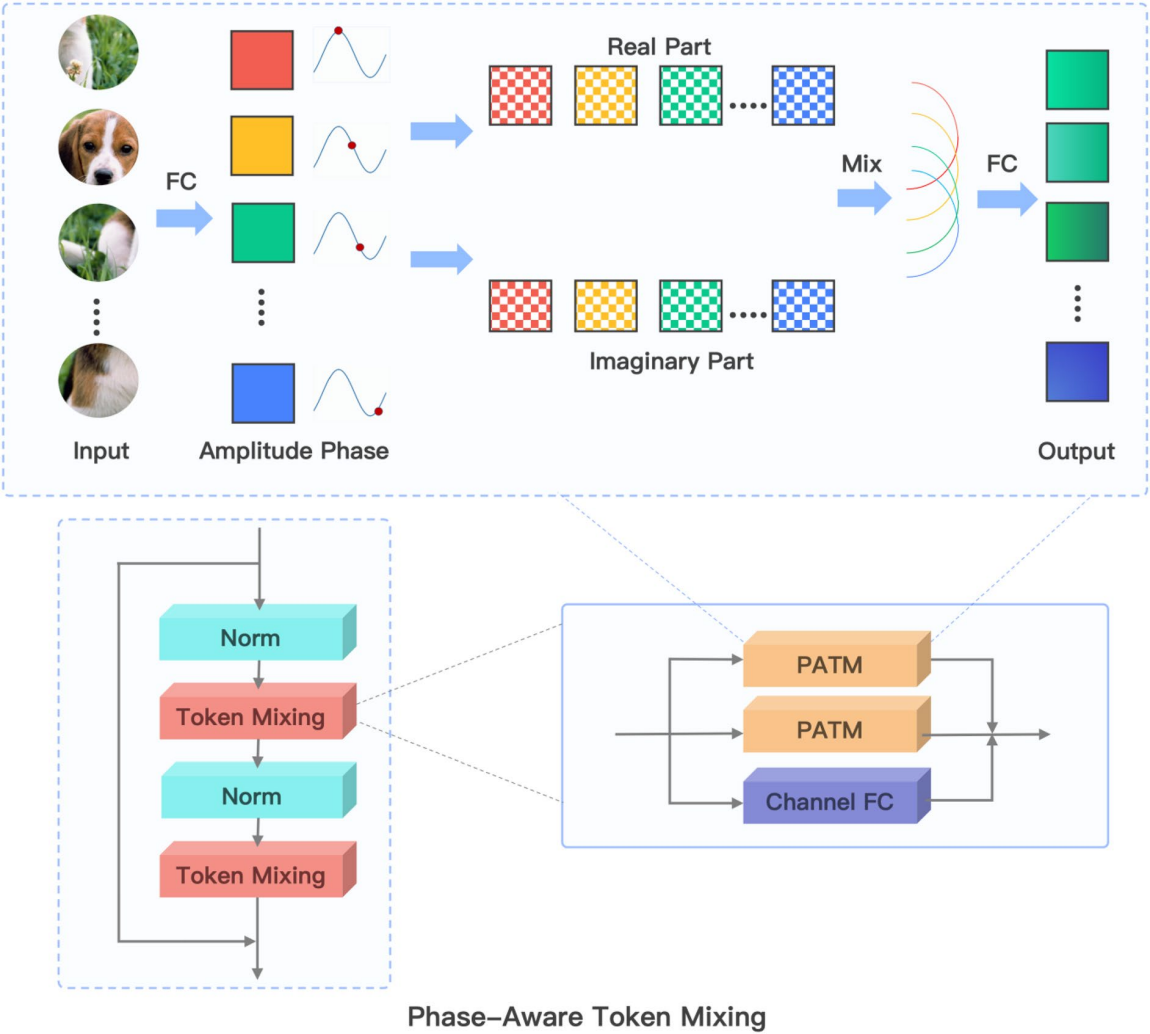
The architecture of the WaveBlock module^29^. The WaveBlock module consists of the Norm module and the token mixing module. The token mixing module consists of a channel FC module and the phase-aware token mixing module, which can decompose the input into basic waves with various phases and amplitudes.

Wave-GANomaly is designed by utilizing GAN-based methods which considering the reconstruction error and latent space deviation between normal samples and abnormal samples. Furthermore, Wave-GANomaly uses multi-feature sets fusion and selection to extend the single feature set ability of capturing the informational features from the input data. The high-level overview of Wave-GANomaly is the same as that of Skip-GANomaly, consisting of a GAN-based generator and discriminator. The detailed Wave-GANomaly is shown in Fig. 1. The generator also adopts the bow-tie design by encoder and decoder. Wave-GANomaly is based on Skip-GANomaly as the design of the high-level overview. In the detailed design, Wave-GANomaly extracts wave-based features by the WaveBlock^29^ (Fig. 2). This module is quantum inspired. It regards arbitrary input as a combination of various de Broglie waves having different phases and amplitudes. The specific model design includes the regularization module (Norm), token-mixing module, and channel MLP. The token mixing module is an original mechanism that is composed of the channel fully-connected (FC) layer and the phase-aware token mixing layer (PATM). The PATM module decomposes input into multiple basic waves with different phases and amplitudes. The WaveBlock module achieves the current state-of-the-art in 2D classification, 2D object detection, and 2D segmentation. Wave-GANomaly fuses feature sets by concatenating wave-based and convolution-based features. The feature selection used by Wave-GANomaly is implemented by the CBAM module^25^ and the SE-Block^35^ module. Regular convolution operators extract features by coupling channel and spatial information. However, the authors of the CBAM module^25^ assume that the decoupled spatial and channel information is better than the coupled features. The decoupled design can learn ‘what’ and ‘where’ to attend in the channel and spatial axes, respectively. The another module used for feature selection is SE-Block^35^, which is an abbreviation of the squeeze-and-excitation (SE) block, with the goal of improving the quality of representations produced by a network. The authors of SE-Block assume that in a given feature map with multiple channels, each channel contribution to capturing the information of input images is not the same. Hence, this module models the interactions between the channels of its features. As a result, the authors of the SE-Block propose a mechanism that allows the network to perform feature recalibration, through which it can learn to use global information to selectively emphasize informative features and suppress less useful ones. Since unsupervised learning models for anomaly detection tasks use only normal samples to train their models, there may exist some probability that these models overfit normal samples. Hence, we used the DropOut module as a way to avoid overfitting^36^. Hence, we add this DropOut module, which is used in many models in deep learning, as a way to avoid overfitting^36^.

Compared to existing GAN-based methods, this work uses multi-feature instead of single feature set. Recently, multi-modal methods have been proposed to extract multi-feature from multi-modal data source. For example, CLIP^31^ utilizes image and text data which obtained a good performance on well-known datasets. In some datasets, the zero-shot inference of CLIP better than supervised model such as ResNet-50 which trained on these datasets. However, the extra feature set may include useless information to the targeted tasks. Hence, directly fuse multi-feature may cause negative impact on the interested task. In this consideration, we added the feature selection block to select good features from multi-feature set. Furthermore, as the number of training parameters increased, the model has higher probability to overfit training data. In this consideration, we added Dropout module to alleviate this problem. Inspired by the wave-based feature set, such as Fourier transform and wavelet transform as feature set in cooperation with various machine learning models have achieved good performance on numerous tasks. However, these wave-based features extracted independently to the model training. In this work, we proposed an end-to-end model which training and feature extracting at the same time.

#### Objective of wave-GANomaly

The objective of the Wave-GANomaly design consists of adversarial loss, contextual loss, and latent loss. The adversarial loss used in this work was proposed by Goodfellow et al.^13^. The model aims to solve the min–max optimization problem.

1 $$\underset{G}{{\text{min}}}\underset{D}{{\text{max}}}{L}_{adv}$$

2 $${L}_{adv}=E\left[logD\left(x\right)\right]+E[{\text{log}}(1-D(x{^\prime})]$$

where *D(x)* represents the discriminator, x represents the input image, and x’ represents the reconstructed image. Equation 2 means that the reconstructed image should be as realistic as possible. The discriminator aims to distinguish an image as real or fake. The second loss we use is the contextual loss (Eq. 3):

3 $${L}_{con}=E|x-{x}{^\prime}|$$

This equation demonstrates that the reconstructed image should be as similar to the original input image as possible. The third loss we used in this work is the latent loss. The latent loss can be calculated by Eq. (4):

4 $${L}_{lat}= E|f\left(x\right)-f\left({x}{^\prime}\right)|$$

where f(x) represents the function that transforms the given input to the latent space.

As a result, the loss takes these three types of loss into consideration (Eq. 5). However, in the different situations, the three losses may not contribute equally. Hence, we design three hyperparameters to let the user tune the corresponding loss to achieve better performance under different situations.

5 $$L={{\lambda }_{adv}L}_{adv}+{{\lambda }_{con}L}_{con}+{{\lambda }_{lat}L}_{lat}$$

#### Abnormal score

To find the abnormal samples during the test, we adopt the abnormal score, which was proposed in GANomaly^15^ and employed in Skip-GANomaly^23^. For a given test image *x*˙, the score considers the error of reconstruction and latent representation and is weighted by a hyperparameter. The details are shown in Eq. (6), where *R(x)* represents the reconstruction error of a given image, and *L(x)* represents the error of latent representation. To normalize the abnormal score, we updated the original abnormal score equation (Eq. 6) by simply normalizing the abnormal score to the range [0,1]. The detailed calculation is shown in Eq. (7).

6 $$A(x) = \lambda R(x)+ (1 - \lambda )L(x)$$

7 $${A}{^\prime}\left(x\right)= \frac{A\left(x\right)-{\text{min}}(A)}{{\text{max}}\left(A\right)-{\text{min}}(A)}$$

## Results

This section consists of the ablation study on the CIFAR-10 dataset, comparisons to the state-of-the-art methods on both the CIFAR-10 dataset and MNIST dataset, and visualization of Wave-GANomaly.

### Ablation study on the CIFAR-10 dataset

**Figure 3:**
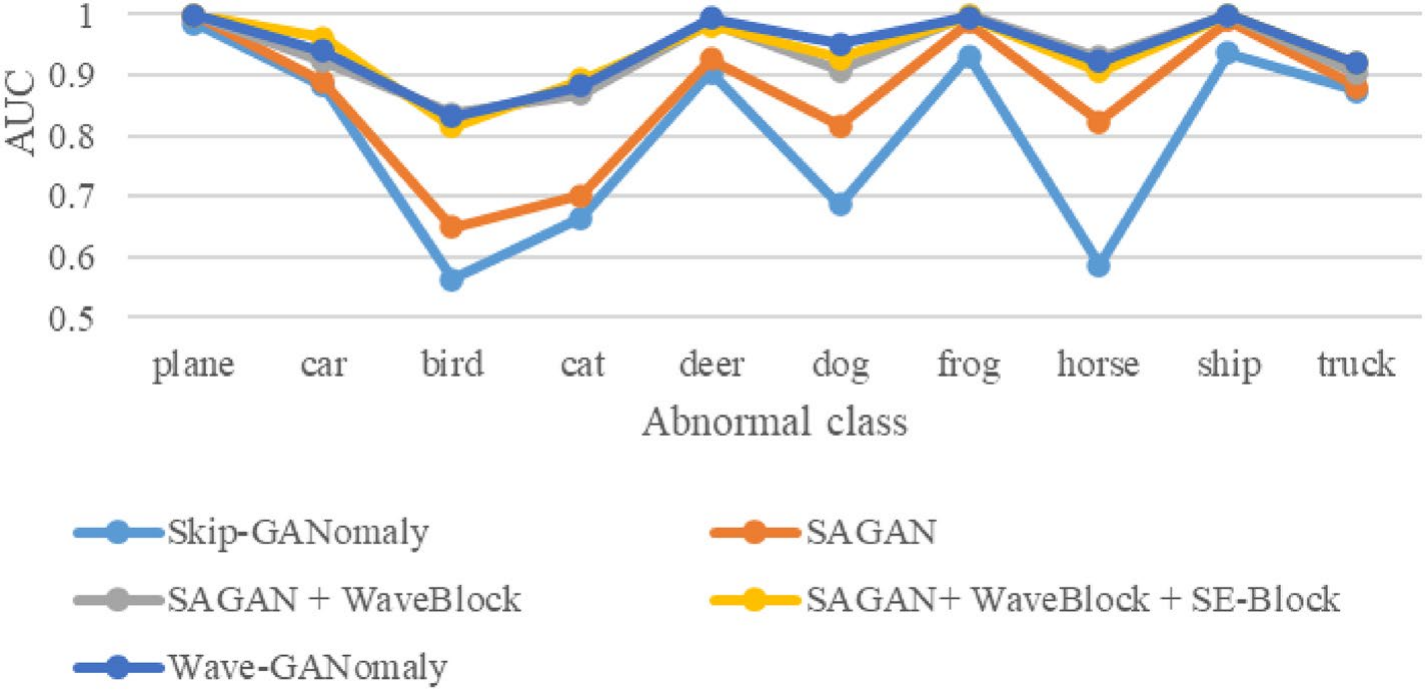
The main results of the ablation study on the CIFAR-10 dataset. This figure integrates the ablation study results of Skip-GANomaly, SAGAN, SAGAN+WaveBlock, SAGAN+WaveBlock+SE-Block, and Wave-GANomaly on the CIFAR-10 dataset. Wave-GANomaly outperforms the compared methods on the CIFAR-10 dataset.

For this section, we conducted comprehensive experiments on the CIFAR-10 dataset. We used Skip-GANomaly as the baseline model and compared the contribution of each Wave-GANomaly module on the dataset. The experiments were conducted in a leave-one-class-out manner. The detailed results of the ablation study are shown in Table 1 and Fig. 3. Experiment A, GANomaly, is the baseline of Skip-GANomaly. This work explores various network designs on a high-level overview. As a result, the design of GANomaly, consisting of a generator and a discriminator, is the best design among models compared to GANomaly. Experiment B, Skip-GANomaly, improved the average AUC from 74.9% to 80.1% by adding skip connections^37^. Experiment C (Experiment B+CBAM) improved the average AUC from 80.1% to 86.6% by adding the CBAM module. Experiment D (Experiment C+WaveBlock) improved the average AUC from 86.6% to 93.5% by adding the WaveBlock module. Experiment E (Experiment D+SE-Block) improved the average AUC from 93.5% to 94.0% by adding the SE-Block module. Experiment F (Experiment E+DropOut) improved the average AUC from 94.0% to 94.3% by adding the DropOut module. Finally, we used Experiment F as the proposed model and further compared it to other state-of-the-art methods on both the CIFAR-10 and MNIST datasets.

**Table 1:** Ablation study on the CIFAR-10 dataset.

Exp.	Method	Plane	Car	Bird	Cat	Deer	Dog	Frog	Horse	Ship	Truck	AVG_AUC
A	GANomaly*	0.968	0.698	0.562	0.604	0.789	0.721	0.902	0.626	0.913	0.707	0.749
B	Skip-GANomaly*	0.985	0.883	0.564	0.664	0.905	0.686	0.931	0.587	0.936	0.873	0.801
C	B+CBAM (SAGAN)	**0.999**	0.889	0.649	0.702	0.926	0.816	0.987	0.823	0.991	0.880	0.866
D	C+WaveBlock	**0.999**	0.922	**0.836**	0.869	0.985	0.907	**0.998**	**0.930**	**0.998**	0.902	0.935
E	D+SE-Block	**0.999**	**0.961**	0.815	**0.893**	0.982	0.928	0.997	0.906	**0.998**	**0.920**	0.940
F	E+DropOut (Wave-GANomaly)	**0.999**	0.939	0.832	0.883	**0.994**	**0.951**	0.995	0.923	**0.998**	**0.920**	**0.943**
G	B+WaveBlock	0.993	0.901	0.671	0.739	0.930	0.750	0.963	0.854	0.977	0.821	0.860
H	B+SE-Block	0.966	0.856	0.629	0.667	0.822	0.823	0.938	0.794	0.982	0.865	0.834
I	B+DropOut	0.994	0.892	0.614	0.659	0.902	0.748	0.973	0.805	0.976	0.868	0.843

The bold values mean the best performance in a specific class.

*indicates the data are using their corresponding codes released on the GitHub page.

### Comparisons to the state-of-the-art methods

#### Comparisons on the CIFAR-10 dataset

**Figure 4:**
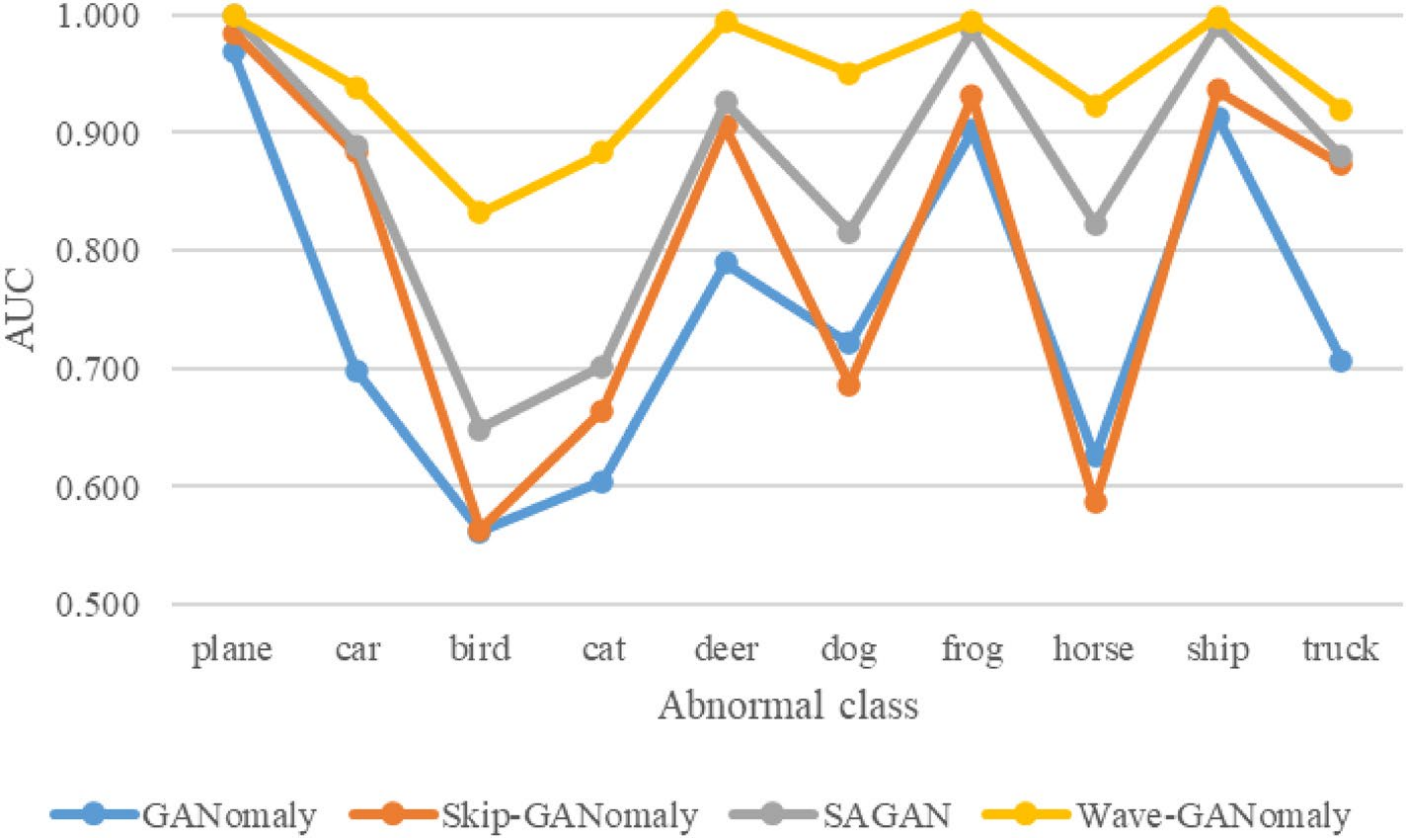
Comparisons of state-of-the-art methods on the CIFAR-10 dataset. This figure integrates the results of the GANomaly, Skip-GANomaly, SAGAN, and Wave-GANomaly results on the CIFAR-10 dataset. Wave-GANomaly outperforms the compared methods on the CIFAR-10 dataset.

Previously, some elegant proposals, including GANomaly, Skip-GANomaly, and SAGAN, have shown good performance on the CIFAR-10 dataset. Hence, this work Wave-GANomaly performs the same experiment setting for fair comparison to these elegant proposals. The detailed results are shown in Table 1 and Fig. 4. GANomaly achieves an average AUC of 74.9% on the CIFAR-10 dataset. Skip-GANomaly achieves an 80.1% average AUC on the CIFAR-10 dataset, and SAGAN achieves an 86.6% average AUC on the CIFAR-10 dataset. The model we propose, Wave-GANomaly (Experiment F), achieves a 94.3% average AUC, which outperforms the compared models on the CIFAR-10 dataset.

#### Comparisons on the MNIST dataset

**Figure 5:**
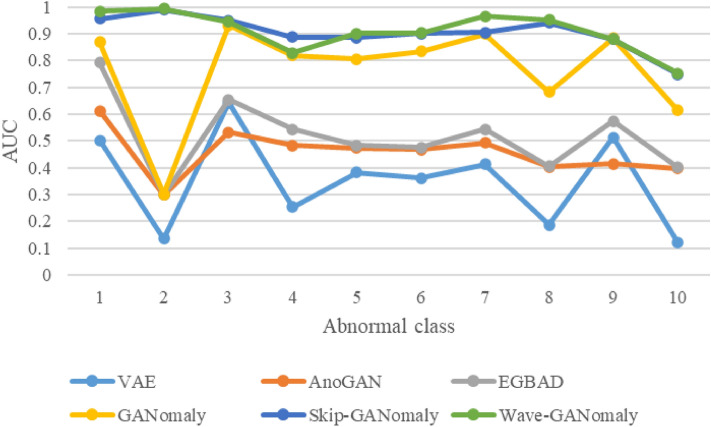
Comparisons of state-of-the-art methods on the MNIST dataset. This figure integrates the results of the VAE, AnoGAN, EGBAD, GANomaly, Skip-GANomaly, SAGAN, and Wave-GANomaly results on the MNIST dataset. Wave-GANomaly outperforms the compared methods on the MNIST dataset.

In this section, we have compared several elegant proposals on the MNIST dataset. The results are shown in Table 2 and Fig. 5. These experiments were also conducted in a leave-one-class-out manner. The VAE method uses a variational autoencoder as an anomaly detector, achieving a 35.1% average AUC on the MNIST dataset. The AnoGAN method achieves an average AUC of 45.7% on the MNIST dataset. The EGBAD method achieves an average AUC of 51.7% on the MNIST dataset. GANomaly achieves an average AUC of 76.4% on the MNIST dataset. Skip-GANomaly achieves an average AUC of 90.4% on the MNIST dataset. Wave-GANomaly achieves an average AUC of 91.0% on the MNIST dataset, outperforming the compared models on the MNIST dataset.

**Table 2:** Comparisons on the MNIST dataset.

Method	0	1	2	3	4	5	6	7	8	9	AVG_AUC
VAE*	0.502	0.136	0.642	0.254	0.383	0.362	0.413	0.187	0.514	0.121	0.351
AnoGAN*	0.611	0.298	0.532	0.482	0.473	0.468	0.492	0.403	0.415	0.398	0.457
EGBAD*	0.792	0.299	0.654	0.543	0.482	0.475	0.543	0.405	0.573	0.401	0.517
GANomaly	0.869	0.301	0.930	0.818	0.805	0.834	0.899	0.682	**0.884**	0.613	0.764
Skip-GANomaly	0.955	0.990	**0.949**	**0.887**	0.886	0.901	0.905	0.940	0.880	0.748	0.904
Wave-GANomaly	**0.983**	**0.993**	0.944	0.828	**0.901**	**0.902**	**0.965**	**0.951**	0.879	**0.753**	**0.910**

The bold values mean the best performance in a specific class.

The Skip-GANomaly experiment results are conducted by its code, which was released on the official GANomaly GitHub page.

*indicates that the data are cited from GANomaly^15^.

#### Results on the LCFC-laptop-defect dataset

In this section, we have compared our method to the GANomaly method. Since other GAN-based methods do not conduct experiments on this dataset. We conduct two experiments to compare Wave-GANomaly and GANomaly. The results are shown in the Table 3. GANomaly achieves 58.9% on the LCFC-Laptop-Defect dataset. Our method achieves 94.1% on this dataset. The results show that the proposed method efficiently improve the performance than the method which only uses single feature set.

**Table 3:** Comparisons on the LCFC-laptop-defect dataset.

Method	AUC
GANomaly	0.589
Ours	**0.941**

The bold values mean the best performance in a specific class.

### Visualization of wave-GANomaly

To test the ability to generate fake images, we use the trained Wave-GANomaly model to compare real and fake images. For simplicity, we test the generator on the CIFAR-10 dataset.

**Figure 6:**
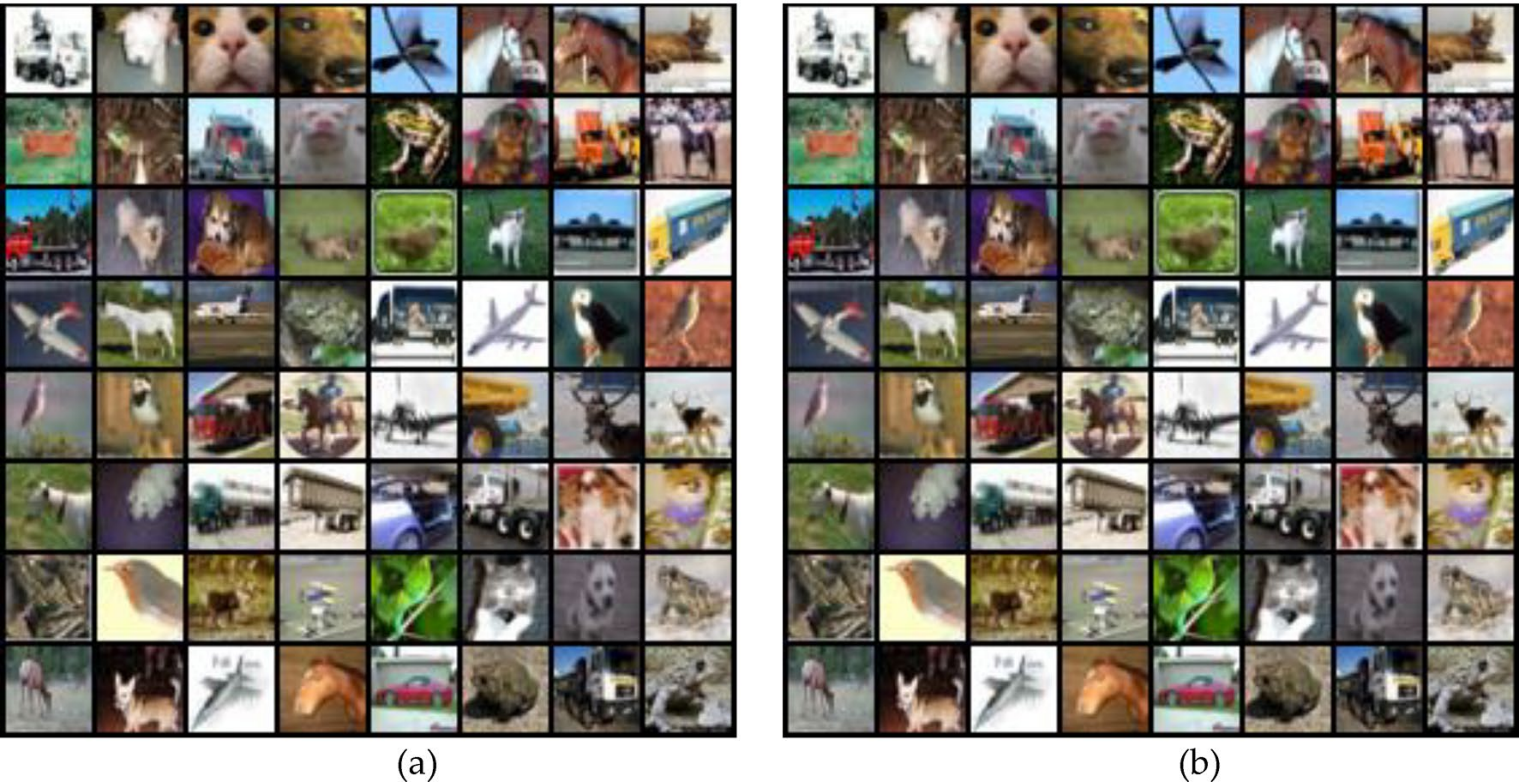
Example of real/fake images on the CIFAR-10 dataset. The fake images are generated by the Wave-GANomaly generator. (**a**) fake images (**b**) real images.

As shown in Fig. 6, (a) represents the fake images and (b) represents the real images from the CIFAR-10 dataset. We found that the fake images generated from the Wave-GANomaly generator are as realistic as the real images. These results provide another aspect to verify whether the generator is trained well instead of using only the classification performance. These results may imply the ability of reconstruction and generation of an input image to achieve good results on the CIFAR-10 dataset. Since the input image size on the CIFAR-10 dataset is small (32 × 32), the small number of parameter values of reconstructed images that needed to be solved is also small. It is easier to reconstruct input images than to input images with large sizes.

## Conclusions and discussion

Compared to the baseline method, Skip-GANomaly, this work (Wave-GANomaly) added the CBAM module, the WaveBlock module, the SE-Block module, and the DropOut module. The added CBAM module is the same as was used in the elegant proposal, SAGAN, and indeed improves the average AUC on the CIFAR-10 dataset. The CBAM module is designed by considering ‘what’ and ‘where’ to capture the more informational features from the foreground instead of the whole image. Wave-GANomaly fuses and selects from the multi-feature sets including the wave-based features extracted by the WaveBlock module and the convolution-based features. The WaveBlock regards a given input as a combination of basic waves with different amplitudes and phases. However, directly fuses multi-feature set increase the computation cost and not all the features are positive impact to the interested task. Hence, we used the SE-Block module used in this work is designed to reweight the feature map in a channel-wise manner. Since the contribution of each channel in a feature map is not the same, reweighting these channels by enhancing more informational features and surpassing the less informational features can improve the performance of anomaly detection. The fourth block, DropOut, is designed by reducing the probability of overfitting. Since the unsupervised learning approach for the anomaly detection task uses only normal samples for the training model, it has a high probability of overfitting the data. As a result, Wave-GANomaly adopted all these modules together and achieved state-of-the-art performance on the CIFAR-10 dataset and the MNIST dataset. For further verify Wave-GANomaly performance on the real-world dataset, we curated the LCFC-Laptop-Defect dataset. In this dataset, our method also has a good performance on this real-world dataset. In this work, a multi-feature model improves the performance on the benchmark datasets and the real-world dataset. However, in the real situation, the high requirements of inference time and performance of industry scenes. The designed model should both accurately and quickly meet these requirements. Hence, we believe that designing a lightweight model for industrial scenes is an important direction.

## Data Availability

The MNIST and CIFAR-10 datasets can be available from PyTorch APIs and their corresponding official websites. The LCFC-Laptop-Defect dataset analyzed during the current study available from the corresponding author on reasonable request.

## References

[1] Han C (2021). MADGAN: Unsupervised medical anomaly detection GAN using multiple adjacent brain MRI slice reconstruction. BMC Bioinform..

[2] Kiran (2018). An overview of deep learning based methods for unsupervised and semi-supervised anomaly detection in videos. J. Imaging.

[3] Chandola V, Banerjee A, Kumar V (2009). Anomaly detection: A survey. ACM Comput. Surv. (CSUR).

[4] Injadat, M., Salo, F., Nassif, A. B., Essex, A. & Shami, A. in *2018 IEEE Global Communications Conference (GLOBECOM)*, (IEEE) 1–6 (2018).

[5] Xu, S. *et al.* PP-YOLOE: An evolved version of YOLO. arXiv:2203.16250 (2022).

[6] Minaee S (2021). Image segmentation using deep learning: A survey. IEEE Trans. Pattern Anal. Mach. Intell..

[7] Hesamian MH, Jia W, He X, Kennedy P (2019). Deep learning techniques for medical image segmentation: Achievements and challenges. J. Digit. Imaging.

[8] Wang, C.-Y., Bochkovskiy, A. & Liao, H.-Y. M. YOLOv7: Trainable bag-of-freebies sets new state-of-the-art for real-time object detectors. arXiv:2207.02696 (2022).

[9] Xie, Q., Luong, M.-T., Hovy, E. & Le, Q. V. in *Proceedings of the IEEE/CVF Conference on Computer Vision and Pattern Recognition*, 10687–10698 (2020).

[10] Pang G, Shen C, Cao L, Hengel AVD (2021). Deep learning for anomaly detection: A review. ACM Comput. Surv. (CSUR).

[11] Chalapathy, R. & Chawla, S. Deep learning for anomaly detection: A survey. arXiv:1901.03407 (2019).

[12] Yu W, Kim IY, Mechefske C (2020). An improved similarity-based prognostic algorithm for RUL estimation using an RNN autoencoder scheme. Reliab. Eng. Syst. Saf..

[13] Goodfellow, I. NIPS 2016 Tutorial: Generative Adversarial Networks Ian Goodfellow arXiv:1701.00160 (2016).

[14] Sabokrou, M., Khalooei, M., Fathy, M. & Adeli, E. Adversarially learned one-class classifier for novelty detection. *IEEE* (2018).

[15] Akcay, S., Atapour-Abarghouei, A. & Breckon, T. P. GANomaly: Semi-supervised anomaly detection via adversarial training (2019).

[16] Zenati, H., Foo, C. S., Lecouat, B., Manek, G. & Chandrasekhar, V. R. Efficient GAN-Based Anomaly Detection Houssam Zenati, Chuan Sheng Foo, Bruno Lecouat, Gaurav Manek, Vijay Ramaseshan Chandrasekhar arXiv:1802.06222 (2018).

[17] An J, Cho S (2015). Variational autoencoder based anomaly detection using reconstruction probability. Spec. Lect. IE.

[18] Zhang, X. *et al.* in *Proceedings of the IEEE/CVF Conference on Computer Vision and Pattern Recognition.* 3914–3923 (2023).

[19] Yang M, Wu P, Feng H (2023). MemSeg: A semi-supervised method for image surface defect detection using differences and commonalities. Eng. Appl. Artif. Intell..

[20] Wang J (2023). Toward surface defect detection in electronics manufacturing by an accurate and lightweight YOLO-style object detector. Sci. Rep..

[21] Zhang, H. & Davidson, I. in *Proceedings of the 2021 ACM Conference on Fairness, Accountability, and Transparency*, 138–148 (2021). 10.1145/3593013.3594102 PMC10661580 37990734

[22] Liu, Z., Zhou, Y., Xu, Y. & Wang, Z. in *Proceedings of the IEEE/CVF Conference on Computer Vision and Pattern Recognition*, 20402–20411 (2023).

[23] Akay, S., Atapour-Abarghouei, A. & Breckon, T. P. Skip-GANomaly: Skip connected and adversarially trained encoder-decoder anomaly detection. *IEEE* (2019).

[24] Liu, G., Lan, S., Zhang, T., Huang, W. & Wang, W. in *2021 IEEE International Conference on Image Processing (ICIP)*, (IEEE) 2468–2472 (2021).

[25] Woo, S., Park, J., Lee, J.-Y. & Kweon, I. S. in *Proceedings of the European Conference on Computer Vision (ECCV)*, 3–19 (2018).

[26] Schlegl, T., Seeböck, P., Waldstein, S. M., Schmidt-Erfurth, U. & Langs, G. in *International Conference on Information Processing in Medical Imaging*, 146–157 (Springer, 2017).

[27] Donahue, J., Krhenbühl, P. & Darrell, T. Adversarial Feature Learning (2016).

[28] Blum, C. W. On the Effectiveness of Neural Networks Classifying the MNIST Dataset (2017).

[29] Tang, Y. *et al.* in *Proceedings of the IEEE/CVF Conference on Computer Vision and Pattern Recognition*, 10935–10944 (2022).

[30] Ronneberger, O., Fischer, P. & Brox, T. in *International Conference on Medical image computing and computer-assisted intervention*, 234–241 (Springer, 2015).

[31] Radford, A. *et al.* in *International conference on machine learning*, (PMLR) 8748–8763 (2021).

[32] Thakkar, V., Tewary, S. & Chakraborty, C. Batch Normalization in Convolutional Neural Networks — A comparative study with CIFAR-10 data Vignesh Thakkar, Suman Tewary, Chandan Chakraborty 1–5 https://ieeexplore.ieee.org/document/8470438.

[33] Deng L (2012). The MNIST database of handwritten digit images for machine learning research [Best of the Web]. IEEE Signal Process. Mag..

[34] Kussul E, Baidyk T (2004). Improved method of handwritten digit recognition tested on MNIST database. Image Vis. Comput..

[35] Hu, J., Shen, L. & Sun, G. in *Proceedings of the IEEE Conference on Computer Vision and Pattern Recognition*, 7132–7141 (2018).

[36] Srivastava N, Hinton G, Krizhevsky A, Sutskever I, Salakhutdinov R (2014). Dropout: a simple way to prevent neural networks from overfitting. J. Mach. Learn. Res..

[37] He, K., Zhang, X., Ren, S. & Sun, J. in *Proceedings of the IEEE Conference on Computer Vision and Pattern Recognition*, 770–778 (2016).

